# The role of individual features of memory and impulsiveness in telling a true or false story in a realistic, clear, and reconstructible way

**DOI:** 10.3389/fpsyg.2023.1173219

**Published:** 2023-07-06

**Authors:** Ida Sergi, Francesca Mottola, Augusto Gnisci, Letizia Caso, Nicola Palena

**Affiliations:** ^1^Department of Psychology, University of Campania “Luigi Vanvitelli”, Caserta, Italy; ^2^Department of Human Sciences, Libera Università Maria SS. Assunta, Rome, Lazio, Italy; ^3^Department of Human and Social Sciences, University of Bergamo, Bergamo, Lombardy, Italy

**Keywords:** metamemory, reality monitoring, cluster analysis, impulsivity, deception

## Abstract

**Objective:**

The aim of the present study was to explore whether there was an interaction effect between such personal aspects and veracity on realism, clarity, and reconstructability of the story.

**Methods:**

A total of 158 participants took part in the experiment and were asked to tell a truth and a lie during an interview (veracity condition). They filled in a questionnaire measuring their metamemory performance and their level of functional and dysfunctional impulsivity. A k-means cluster analysis on metamemory and impulsivity was conducted, and three clusters were obtained: controlled-memory inefficient, controlled-memory efficient, and impulsive-average memory.

**Results:**

The results showed that participants scored higher on all three reality monitoring criteria when telling the truth than when lying. Further, a cluster membership by veracity interaction for realism was also significant, but when telling the truth, there was no difference between clusters in terms of realism used in the explanation. Follow-up analyses showed that, when lying, the level of realism in the story was significantly higher for people belonging to the cluster “impulsive-average memory” than for people belonging to the cluster “controlled-memory efficient”, a result that seems to indicate that people with good memory and can control dysfunctional impulsivity have more difficulties when lying.

**Conclusions:**

Research has shown that realism, clarity, and reconstructability of the story, all part of reality monitoring, can be useful to assess veracity. Generally, truth tellers obtain higher scores on all three variables than liars, but there is some variability across individuals owing to their personal characteristics. Metamemory and impulsivity also play a role in deception. From the implications of the results, the limitations of the study and suggestions for future research are also provided.

## 1. Introduction

Initially, the focus was on the search for specific verbal and non-verbal cues to deception (Granhag et al., 2015). However, although there are some cues that are statistically associated with lying, effect sizes are still small to moderate (Hauch et al., 2017; Palena et al., 2021b; Vrij et al., 2021). This happens for several reasons. First, researchers focused on truth cues and overlooked lie cues (Vrij et al., 2022). Second, humans are generally not accurate in lie deception, and indeed, our mean accuracy is ~54% (Bond and DePaulo, 2006, 2008). Third, interpersonal differences play an important role (Caso et al., 2018). For these reasons, a great amount of research at present focuses on the development of effective interviewing approaches that can maximize the amount of collected information and enhance differences between truth telling and lying. One example to deal with such differences is the baseline approach (Vrij, 2016), which builds on the idea that, if an investigator has a baseline reference of how someone behaves and talks when telling the truth, then deciphering if someone is lying should be easier than not having a baseline. Another and more recent approach is the application of person-centered methodologies in interviewing settings (Palena and Caso, 2021). Briefly speaking, the more common variable-centered approach assumes that the effect under investigation is the same across individuals. On the contrary, one of the pillars of the person-centered approach is that an effect is not the same for everyone. Indeed, the person-centered approach assumes that people can be grouped into specific subpopulations (often called clusters or profiles) through data-driven procedures. Consequently, people belonging to the same subpopulation (i.e., cluster or profile) are more similar to each other in the pattern of scores of the variables taken into account than people belonging to different subpopulations. It follows that whatever effect is being studied can be moderated by group membership. To provide an example, a researcher might be interested in studying the effect of different teaching methods on students' performance and assume that the personality profile (group membership) of their participants moderates such a relationship.

To the best of our knowledge, there are only a few examples of the applications of this approach in lie detection research. For example, Palena et al. (2021a) analyzed participants' scores on the five factors of personality, moral disengagement, and their perceived cognitive load when lying and obtained four profiles showing different patterns in such variables (e.g., one profile was characterized by high extraversion and high perceived cognitive load when lying, whereas another profile showed high extraversion but low perceived cognitive load when lying). They then ran additional analyses and found that profile membership was associated with lying behavior. In essence, profile membership predicted lying behavior. Similarly, Palena et al. (2022) obtained profiles starting from participants' scores on the six factors of personality, Machiavellianism, and moral disengagement and found that profile membership was associated with, among others, lying ability and lying frequency. These studies indicated that lie detection research might benefit from the application of person-centered approaches that, although new to the topic of lie detection, are well-known in other research areas (Palena and Caso, 2021). Indeed, given the high variability between individuals in lying behavior, person-centered approaches provide a solid psychometric ground to deal with interpersonal differences.

Several instruments for the detection of verbal lies have been used in the literature, such as the Statement Validity Assessment (SVA) and the Scientific Content Analysis (SCAN) (Vrij, 2008). However, reality monitoring (RM) is scientifically more robust because it refers to the cognitive processes that discriminate between perceived events and imagined events. The assumption is that memories based on real experiences differ in quantity and/or quality from memories based on fiction (Johnson and Raye, 1981). As a result, in the early 1990s, the RM approach was widely accepted as potentially one of the most efficient tools for verbal lie detection (Vrij, 2000; Sporer, 2004; Vrij et al., 2004; Masip et al., 2005). The first clear and comprehensive operationalization of the RM criteria can be observed in the study by Vrij (2000), who proposed eight criteria: Clarity, Perceptual Information, Spatial Information, Affective Information, Reconstructability, Realism, Temporal Information, and Cognitive Operations. The RM criteria have been applied to lie detection research, and researchers found them to discriminate truth telling from lying with up to 70% accuracy rates (Vrij, 2015; Hauch et al., 2017).

According to the RM model, the memory of an actual event has more perceptual, has more contextual information, has more affective information, sounds clearer, is more realistic, and is reconstructable (Johnson and Raye, 1981). For this reason, it is easier to recall and retrieve the memory of an actual event (i.e., everything that is outside us) than an invented one (Posner and Warren, 1972; Brown, 1975; Posner and Snyder, 1975; Hasher and Zacks, 1979). Recently, Besken (2018) examined the relationship between deception and memory while also assessing the metamemory of liars and truth tellers. Participants provided correct (truthful) or incorrect (lie) answers to a series of general knowledge questions and later estimated their confidence that they would remember their responses on a subsequent test. This study showed that people predicted that they would remember truthful responses better, but, in reality, they recalled more lie responses, so people overestimated their ability to accurately source their memory. These results are particularly surprising given that truth experiences are often better remembered than lied experiences (Vieira and Lane, 2013; Dianiska et al., 2019; Dianiska and Meissner, 2022). Starting from this, the aspect we believe is interesting to understand is what people think about their ability to remember their lies (and truths) over time. In the present experiment, we focused on verbal cues to deception and truth and just examined them through reality monitoring.

However, most of the criteria of the RM are impractical, as this would require that the interviewer counts them in real time, which is an impossible task (Vrij et al., 2022). Further, the countable details of the RM are culture dependent and, likely, also context dependent (e.g., Taylor et al., 2015). For these reasons, we only focused on the three impression cues of the RM: realism, reconstructability of the story, and clarity, which are the general RM criteria and are used to understand the truthfulness of a story.

The second aspect we decided to investigate is the features of metamemory (MM) because they could be involved in the deceptive process. MM refers to people's knowledge about learning and memory processes in general and to the assessment (monitoring) and regulation (control) of these processes as they occur (Flavell, 1971). This cognitive process involves awareness of one's own resources and limitations. The ability to correctly and realistically assess one's skills, abilities, and efficiency, in terms of accuracy, precision, appropriateness, and speed of execution, results in better control and adaptability of the individual to the demands of the environment. Despite this, when asked to recall information, people tend to display two biases: similar memory predictions for different time intervals and overconfidence in memory performances. These errors constitute stability bias (Liu, 2019). Recent research (e.g., Harvey et al., 2017) suggests that liars were unable to precisely tune the amount of detail disclosed to simulate the effects of forgetting over time associated with genuine memory. The liar's insensitivity to delayed manipulation suggests a stability bias affecting their verbal behavior. Consequently, liars are more prone to metacognitive errors when lying after extended intervals (Harvey et al., 2017). To measure this complex aspect of metacognition, the MM questionnaires contain several subscales to capture different features of memory (Gopi and Madan, 2022); for this study, we took into account four aspects of the memory functioning based on self-appraisal: frequency of memory failures, severity of memory failures, changes of memory performance over time, and the use of memory facilitating strategies. The frequency and the seriousness of memory failures refer, respectively, to how often memory mistakes occur for specific situations (Bennett-Levy and Powell, 1980; Sehulster, 1981; Zelinski and Gilewski, 2004) and how serious one perceives their memory failure. Instead, the changes in memory performance over time refer to a subjective assessment of own mnestic abilities compared with earlier periods of their life. Finally, the last characteristic refers to the use of facilitating memory strategies, including internal memory aids such as mnemonics and external aids such as calendars (Dixon and Hultsch, 1983; Bouazzaoui et al., 2010) or mental repetition of items.

The third aspect we decided to investigate concerns a personality characteristic: impulsivity. Impulsivity may be defined as the tendency to act on immediate urges, either before the consideration of possible negative consequences or despite the consideration of likely negative consequences (DeYoung and Rueter, 2016). Dickman (1990) conceptualized impulsivity as a multi-dimensional construct and is comprised of two factors. Functional impulsivity refers to the tendency to make quick decisions with advantageous outcomes. In contrast, dysfunctional impulsivity refers to the tendency to act without forethought in situations in which this behavior is not advantageous. In general, when considering the relationship between impulsivity and lying, research studied only functional impulsivity. Indeed, some studies have found that (functional) impulsivity is related to lying. Makowski et al. (2021) have found that individuals with difficulties in cognitive control tend to have a higher lying frequency, and this pattern was found across different measures, such as impulsivity, emotion regulation deficits, and disinhibited behavior. Kumari (1996) showed that a high score on the lie scale was associated with a higher score on impulsivity. Consequently, we can deduce that lying is associated with impulsivity. When people truthfully describe or deny an action, they can rely on their memory of the experience to process a response (Dianiska and Meissner, 2022). In contrast, lying takes longer to produce a response (Suchotzki et al., 2017) and is more cognitively demanding (Vrij et al., 2008). Therefore, when dysfunctional impulsivity is involved, lying should result in a more confusing, contradictory, or unrealistic report of the events. Instead, as far as we know, no study ascertained the role of functional impulsivity in the lying process. Hypothetically, people with functional impulsivity should be able to tell a coherent and clear story of what occurred even when lying.

Building on the above literature, we expected that the effect of veracity on source monitoring would be moderated by participants' cluster membership. In particular, we expected that the difference between truth telling and lying on source monitoring scores would be higher for participants belonging to a cluster characterized by high impulsivity and worse meta-memory than for participants belonging to a cluster showing an opposite trend.

## 2. Methods

The present experiment is based on a dataset previously used for the study of the effect of suspicion and liars' strategies on reality monitoring (Gnisci et al., 2010). However, in the present study, we took a different look at the data by focusing on memory and personality-related variables (see below) and by employing a person-centered approach. Such a statistical approach has rarely been used in deception research experiments (Palena et al., 2021a, 2022) but has the advantage of accounting for interpersonal differences (Caso et al., 2018; Palena and Caso, 2021).

### 2.1. Participants

In total, 158 participants (≈ 65% females) took part in the experiment. Their mean age was *M* = 21.90 (*SD* = 2.80). All the participants were Italian students; they were recruited in the Department of Psychology or Biology of the university now labeled University of Campania “Luigi Vanvitelli”. Their participation was voluntary, and they did not receive any incentive for the participation. Multivariate observed power ranged from 0.76 to 1.

### 2.2. Variables and instruments

Metamemory was measured via the Memory Functioning Questionnaire (MFQ; Gilewski et al., 1990). The MFQ consists of 64 items rated on a 7-point Likert scale (1 *vs*. 7 = *Gives me big vs. not at all problems*) and includes four scales. The first scale is named Frequency of Forgetting and includes ratings of how frequently forgetting occurs. This scale consists of 28 items divided into four subscales: the General Rating, Frequency of Forgetting, Frequency of Forgetting When Reading, and Remembering Past Events. The second scale is called Seriousness of Forgetting, which consists of 18 items ratings of memory failures. Retrospective Functioning, the third scale, includes ratings of change in memory ability relative to 5 points earlier in life. The last scale, Mnemonics Usage, consisted of items from the frequency with which eight specific mnemonics are used. Higher scores suggest a more positive evaluation of self-perceived memory functioning and less frequent use of memory aids or strategies. We adopted the Italian version of MFQ (Pedone et al., 2005).

Impulsivity was measured via the Dickman's Impulsivity Inventory (DII; Dickman, 1990) that is a 23-item self-report measure that distinguishes between two types of impulsivity: functional and dysfunctional. Functional impulsivity is the tendency to make quick decisions when such decisions are appropriate for the situation, and 11 items assess this type of impulsivity (e.g., “People have admired me because I can think quickly.”). Dysfunctional impulsivity is the tendency to make quick decisions in contexts when such decisions are not adaptive and 12 items assess this type (e.g., “I often get into trouble because I don't think before I act.”). Items were answered on a 5-point Likert scale (1 *vs*. 5 = *Does not describe me at all vs. Describes me completely*). Higher scores indicate higher levels of functional and dysfunctional impulsivity characteristics. Because an Italian version of the instrument was not available, the questionnaire was translated from English to Italian.

The original dataset also included measures of both verbal content and non-verbal behavior in the perception of lying. Among them, transcripts were coded via reality monitoring criteria (Johnson and Raye, 1981) by three coders^1^ that received training from an expert coder and were blinded about the experimental procedure and the study objectives.

For the present experiment, we were only interested in three criteria of the reality monitoring: realism, clarity, and reconstructability of the story, which were coded on a 3-point scale, ranging from 0 to 2 (0 = *Absent*, 1 = *Present*, 2 = *Strongly Present*). The average inter-rater reliability, measured via Cronbach's alpha, was 0.91 for the first, 0.77 for the second, and 0.93 for the third, indicating good agreement between coders.

### 2.3. Procedure

Once the participants arrived at the site of the experiment, they were welcomed by the experimenter. The experimenter opened a backpack and asked the participant to take a pencil case out of the backpack and observe its content. The experimenter told the participants that the experiment aimed at examining how good people are at telling lies. The participants were also informed that they would be interviewed twice about the objects that were in the backpack and the person with whom they interacted with. The participants were told that, for one interview, they would be asked to be honest, and for the other interview, they would be asked to lie about what they saw in the backpack and the interaction with the experimenter, adding that in neither case the interviewer knew whether the participants were honest or not. The experimenter told them in which of the two interviews they should lie. The participants were then left alone and were given time to prepare for the interview. Then, a first interviewer entered the room and interviewed the participants and then left the room when the interview was over. Then, a second interviewer entered the room and did the same as the first interviewer. The interviews were structured and consisted of 12 questions. The analyses presented in this study only focused on the weak suspicion section of the interview (10 questions), during which the interviewer showed a weak suspicion toward participants' sincerity (full description of the experimental procedure in the original paper: Gnisci et al., 2010). This study was conducted in conformity with the Declaration of Helsinki.

### 2.4. Statistical analyses

Here, we will provide basic information on the statistical analyses we used. More details will be provided in the results.

First, we performed a cluster analysis on the six variables, four regarding memory and two regarding impulsivity. Once the clusters were obtained, they were put in relation with the RM variables via a multivariate analysis of variance where the cluster membership was a between-subjects factor, veracity was a within-subjects factor, and realism, clarity, and reconstructability of the story RM criteria were the dependent variables. *Post-hoc* tests for main effect and interactions were executed by comparing groups with Bonferroni correction for multiple testing.

## 3. Results

### 3.1. Cluster analysis for individual profiles

For the k-means cluster analysis on the memory and impulsivity variables, we assessed the normality of the data and Hopkin's H via the R package *Performance* to assess whether the data were suitable for clustering. Further, a method agreement procedure based on the aggregation of 28 different algorithms was used to explore the optimal number of clusters to retain. The maximum number of iterations for convergence was left at the default value of *n* = 1,000. The six variables used for the cluster analysis were converted to z-scores before analyzing the data. Totally, within-clusters sum of squares and between-clusters sum of squares were reported to describe the variability within and between clusters. Normality was deemed to be present if skewness did not exceed |2| and kurtosis did not exceed |7| (West et al., 1995), whereas Hopkin's H below 0.5 was deemed as indicative of data suitable for clustering (Lüdecke et al., 2019, 2020; Makowski et al., 2021).

**Figure 1:**
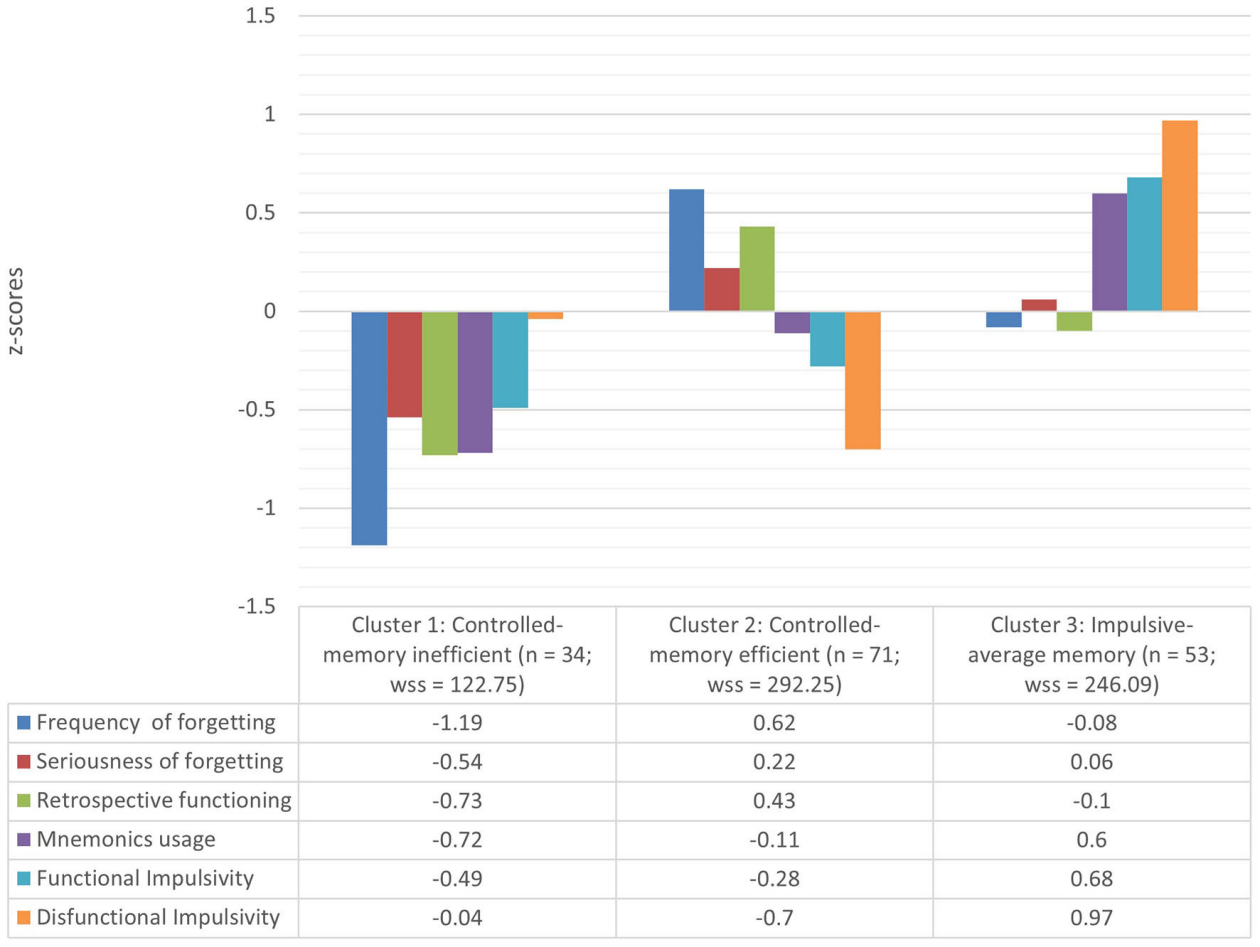
Cluster descriptives. Total variance explained 29.82%; BSS (Between Sum of Squares) = 280.91; TSS (Total Sum of Squares) = 942 (WSS = Within Sum of Squares).

All variables were normally distributed (Skewness_MAX_ = −1.35; Kurtosis_MAX_ = 1.02) and Hopkin's H was 0.39, indicating that the data were suitable for cluster analysis (Lüdecke et al., 2019; Makowski et al., 2021). The analysis also showed that 8 out of 28 algorithms (28.5%) supported the presence of three clusters. Within-clusters, between-clusters, and total sum of squares and z-scores of the three clusters are reported in Figure 1.

The first cluster was characterized by low scores on all the scales of metamemory and functional impulsivity, whereas dysfunctional impulsivity was about the grand mean. This cluster was the one with lower variability between its members. Cluster 1 was labeled “controlled-memory inefficient” and appears to be the worst group in terms of the combination of memory and impulsivity features out of the three.

The second cluster was characterized by high scores on three scales of metamemory, namely frequency and seriousness of forgetting and retrospective functioning, about average scores on the fourth scale of metamemory (mnemonic usage) and low scores on both impulsivity scales, particularly on the dysfunctional one. Therefore, this group was labeled “controlled-memory efficient” and appears to be the best group in terms of the combination of memory and impulsivity.

The third cluster was characterized by average scores on three scales of metamemory, namely frequency and seriousness of forgetting and retrospective functioning, high scores of mnemonic usage, and high scores on both scales of impulsivity. This cluster was labeled “impulsive-average memory”. This is an intermediate group in terms of memory and impulsivity features.

### 3.2. Is there an effect of individual profiles and of telling the truth/lying on real monitoring?

A 3 (clusters; between-subjects) X 2 (veracity: truth *vs*. lies; within-subjects) MANOVA was conducted on the RM scores of realism, reconstructability of the story, and clarity as dependent variables. At a multivariate level, all three effects were statistically significant: cluster, *F*_(6, 308)_= 2.35, *p* = 0.03, ηp^2^ = 0.04, veracity, *F*_(3, 153)_ = 35.74, *p* < 0.001, ηp^2^ = 0.41, cluster by veracity interaction, *F*_(6, 308)_ = 2.15, *p* = 0.048, ηp^2^ = 0.04.

At a univariate level, none of the three RM scores were statistically different between clusters (all *p*s > 0.08). There was a significant main effect of veracity for realism, *F*_(1, 155)_ = 76.31, *p* < 0.001, ηp^2^ = 0.33, reconstructability of the story, *F*_(1, 155)_ = 50.36, *p* < 0.001, ηp^2^ = 0.24, and clarity, *F*_(1, 155)_ = 13.18, *p* < 0.001, ηp^2^ = 0.08. Participants obtained higher scores on realism (*M* = 1.78, *SD* = 0.42), reconstructability of the story (*M* = 1.34, *SD* = 0.67), and clarity (*M* = 1.44, *SD* = 0.55) when telling the truth than when lying (realism, *M* = 1.23, *SD* = 0.69; reconstructability, *M* = 0.96, *SD* = 0.63; reconstructability clarity, *M* = 1.27, *SD* = 0.58).

**Figure 2:**
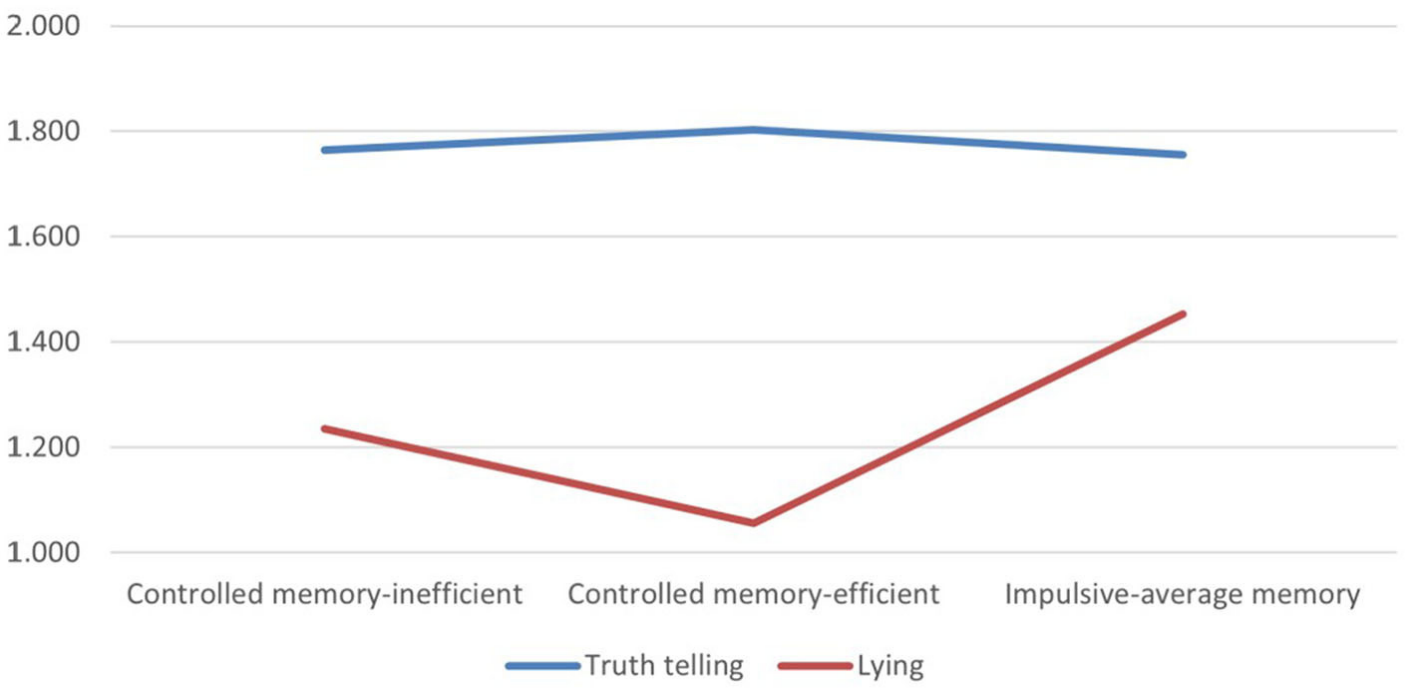
Means of Realism scores for truth telling and lying in the three different clusters (Cluster 1: Controlled-memory inefficient; Cluster 2: Controlled-memory efficient; Cluster 3: Impulsive-average memory).

Of the three possible interaction effects, only the one for the variable realism was significant, *F*_(2, 155)_ = 5.75, *p* < 0.01, ηp^2^ = 0.07. Table 1 shows the average scores for this interaction effect (Figure 2).

**Table 1:** Realism means and standard deviations for truth telling and lying split by cluster membership.

	**Cluster 1: Controlled-memory inefficient**	**Cluster 2: Controlled-memory efficient**	**Cluster 3: Impulsive-average memory**
Realism truth telling *M*(*SD*)	1.77 (0.43)	1.80 (0.40)	1.76 (0.43)
Realism lying *M*(*SD*)	1.24 (0.78)	1.06 (0.63)	1.45 (0.64)

For the interaction, we executed the *post-hoc* tests with Bonferroni correction across clusters within each condition (truth telling *vs*. lying). Given that we executed six comparisons in all (three within each condition), the adjusted threshold for an alfa level of 0.05 was 0.0083 (that is 0.05/6). When telling the truth, there was no difference between clusters in terms of realism used in the explanation (minimum *p* = 0.528). When lying, Cluster 1 was not significantly different from the two other clusters (*p*= 0.200 and *p* =0.140); however, the level of realism in the story was significantly higher in Cluster 3 with respect to Cluster 2 (*p* = 0.001). Therefore, people with good memory who can control dysfunctional impulsivity seem to have more difficulties in lying, because they use stories that seem less realistic and therefore less believable.

## 4. Discussion

The first contribution of this study is to have identified three profiles of individual features, based on two strongly related aspects as metamemory and impulsivity and their sub-dimensions. In terms of memory features and control of impulses, the most functional profile is the one presented as Cluster 2. This group has, in general, optimal features of memory with good control of impulses, which prevents them from realizing that their actions lead to negative consequences. In an intermediate position is Cluster 3, which performs at an average level on three features of metamemory but makes wide use of mnemonic techniques associated with high functional and dysfunctional impulsivity. A possible key could be that the high use of mnemonic techniques in this group could be a kind of antidote to their general, and particularly dysfunctional, impulsivity. The less efficient profile is Cluster 1, with low scores on all the aspects of metamemory and functional impulsivity, whereas dysfunctional impulsivity was about the grand mean.

As far as the effect of the three profiles and the veracity condition on the aspects of RM, we found that the profiles did not have an effect, but that veracity did. Indeed, in truth-telling condition, the participants told a more clear, vivid, reconstructible, and realistic story than when they lied. Moreover, we found an interaction effect of profiles and veracity on realism. Particularly, an understanding that realism always remains greater when telling the truth, when lying, those with good memory, and those with a good control of dysfunctional impulsivity tell a less realistic story than people with average memory and highly functional and dysfunctional impulsivity (i.e., Cluster 3). Therefore, our data show that people very effective in memory and control of dysfunctional impulses may tell less realistic stories when lying, probably because, in lying, they do not have a real memory of the event to remember. People with average memory and high impulsivity, but provided with a good capability of mnemonic use, instead, may provide a more realistic performance when lying. Recent studies (Besken, 2018; Dianiska and Meissner, 2022) have found that individuals who were aware of their own memory inaccuracies were more successful at lying than those who overestimated their memory abilities. Our findings add further information that helps to delineate the role of metacognition in influencing our ability to deceive. The result whereby no main effect was found for cluster membership could be because, rather than directly influencing RM scores, cluster membership act as a moderator for the within-subjects effect of veracity. In essence, cluster membership affects the difference that the same individual shows when telling the truth vs. lying.

Research on lie detection shows that relying on verbal behavior is more effective than relying on non-verbal behavior (Vrij, 2015). When looking at speech, truth telling is often associated with higher scores on truth criteria, such as realism, clarity, and reconstructability of the story than lying (Masip et al., 2005). However, sometimes, there might be no difference at all or sometimes, liars might report a higher frequency of truth criteria than truth tellers. This is likely due to the fact that contextual factors play an important moderating role, and one such factors is culture. For example, research showed that lying is associated with a decreased reporting of spatial information for white British and Arabian people but an increased reporting for North African and Pakistani populations (Taylor et al., 2015). In our research, all the participants were Italian students.

The personal characteristics of each individual also play an important role, and this might explain why some people are more transparent than others (Levine, 2010). Specifically, the results obtained in this study showed that impulsivity (both functional and dysfunctional) can play an important role in telling truth or lying. These results are consistent with those of other studies that have shown both a relationship between Machiavellianism and the tendency to lie (Palena et al., 2022) and a relationship between functional and dysfunctional impulsivity and Machiavellianism (Jones and Paulhus, 2011). It follows the principle that, to enhance the chances to detect lies, the personality and nuances of the interviewee should be accounted for. In the same vein as Palena et al. (2021a, 2022), we explored the effect of individual profiles on lying and found an interaction effect between veracity and profiles (which in our case were obtained starting from meta-memory and impulsivity). This suggested that the effect of veracity might not be constant across individual profiles, which in turn supports the idea that the search for cues to truth/deception should be tailored based on the interviewee profile, although this will be a difficult task due to the huge amount of other contextual variables at play and on the difficulty to decide what variables should be detected to obtain the profiles. Nonetheless, this is a possible new research line for future research. In this perspective, identifying additional personality traits that could be linked to truth-telling or lying behavior would be of interest. We hope that the findings of this study will encourage investigators to pay attention not only to non-verbal behaviors when attempting to detect deceit but also to verbal cues, using verbal veracity assessment tools, such as RM. Moreover, we hope that they will also pay attention to interviewees' individual characteristics and put them in relation to possible interrogation strategies as support to their work.

Although we obtained interesting results, our experiment had some limitations. First, all participants belonged to the same culture; thus, the generalizability of our results can be limited. Second, we only focused on three verbal criteria and did not account for non-verbal behavior, omitting some information that could have supported verbal cues in the detection of lying. Third, we did not employ any specific interviewing technique, and this could have affected the accuracy of lie detection. Indeed, a study (Mac Giolla and Luke, 2021) showed that, for example, the Reality Interviewing protocol (Bogaard et al., 2019) can detect lies with almost 76% accuracy. Fourth, the number of participants within each cluster was unbalanced. Although, commonly, clusters differ in the number of members, this unbalance might have affected the results. Fifth, we should have done a manipulation check to understand whether participants really behaved according to the condition in which they were located. Future research should thus take into account these limitations, for example, by studying if the relationship between cluster membership and veracity on RM criteria is moderated by culture, if the results change when focusing on other verbal cues such as those from the CBCA, and if the application of a specific interviewing protocol affects the results. Further, a larger sample size is desirable so that it would be possible to randomly select participants from within each cluster to reach equal sample sizes.

## Data availability statement

The raw data supporting the conclusions of this article will be made available by the authors, without undue reservation.

## Ethics statement

Ethical review and approval was not required for the study on human participants in accordance with the local legislation and institutional requirements. Written informed consent from the patients/participants or patients/participants legal guardian/next of kin was not required to participate in this study in accordance with the national legislation and the institutional requirements.

## Author contributions

IS contributed to the conception and design of the study, collected data, organized the database, and wrote the first draft of the manuscript. NP performed the statistical analysis, wrote the first draft of the manuscript, and revised substantial parts of the manuscript. FM wrote the first draft of the manuscript and wrote sections of the manuscript. AG contributed to the conception and design of the study and performed the statistical analysis. LC contributed to conception and design of the study and organized the database. All authors contributed to manuscript revision, read, and approved the submitted version.

## Footnotes

^1^One coder coded all transcripts, whereas the other two coders coded 30% of the transcripts.
